# Case Report: Cerebrospinal fluid eosinophilia in adult autoimmune GFAP astrocytopathy

**DOI:** 10.3389/fimmu.2025.1659720

**Published:** 2025-10-10

**Authors:** Yuting Hou, Chunmei Zhao, Xu Wang

**Affiliations:** ^1^ Department of Cerebrospinal Fluid Laboratory, The General Hospital of Ningxia Medical University, Yinchuan, China; ^2^ Department of Neurology, The General Hospital of Ningxia Medical University, Yinchuan, China

**Keywords:** glial fibrillary acidic protein (GFAP), cerebrospinal fluid, cytologic evaluation, eosinophil, case report

## Abstract

A 30-year-old Chinese male diagnosed with autoimmune glial fibrillary acidic protein (GFAP) astrocytopathy presented with cerebrospinal fluid (CSF) eosinophilia. Magnetic resonance imaging (MRI) revealed no significant abnormalities; however, CSF analysis demonstrated the presence of GFAP-immunoglobulin G (IgG) antibodies. Notably, the CSF showed marked eosinophilia, which declined following methylprednisolone therapy and was accompanied by clinical improvement. The proportion of eosinophils in the CSF of this patient exceeded previously reported levels in cases of autoimmune GFAP astrocytopathy. These findings suggest a potential relationship between eosinophil infiltration and the pathogenesis of autoimmune GFAP astrocytopathy, indicating that eosinophils may contribute to disease progression.

## 1 Introduction

Autoimmune glial fibrillary acidic protein (GFAP) astrocytopathy is an inflammatory autoimmune disorder first described in 2016 as a form of meningoencephalitis, with or without myelitis (1). The etiology and pathogenetic mechanisms underlying autoimmune GFAP astrocytopathy remain unclear. However, the presence of GFAP-immunoglobulin G (IgG) in the cerebrospinal fluid (CSF) and/or serum serves as a specific biomarker for this condition, and detection of GFAP-IgG antibodies in CSF is significantly higher than in serum (2). Cytologic evaluation of CSF is an essential component of the neurologist’s diagnostic workup and represents a rapid and cost-effective screening method for various central nervous system (CNS) disorders (3, 4). CSF lymphocytic pleocytosis is commonly observed in the majority of cases of autoimmune GFAP astrocytopathy (1, 5–7), and several studies have characterized the cellular composition of CSF by differentiating between mononuclear and multinucleated cells (8, 9). Eosinophils in the CSF represent an uncommon finding and are predominantly associated with parasitic infections; however, they have also been reported in certain neuroinflammatory disorders. To the best of our knowledge, eosinophils in CSF have been documented in only a limited number of cases of autoimmune GFAP astrocytopathy (10, 11).

Here, we present the case of a 30-year-old male patient who developed fever and headache. Brain magnetic resonance imaging (MRI) did not reveal any significant abnormalities, and neurological examination was unremarkable except for neck stiffness. Cell-based assays confirmed the presence of GFAP-IgG antibodies in the CSF, whereas serum testing was negative. Notably, CSF cytological analysis revealed marked eosinophilia, with the maximum eosinophil percentage reaching 22% and the eosinophil count amounting to 24.2 cells/mm³—both values significantly exceeding those previously reported in cases of autoimmune GFAP astrocytopathy. Following treatment with methylprednisolone, CSF eosinophil levels declined, which corresponded with clinical improvement. The peripheral eosinophil count remained within normal limits throughout the course of the disease.

## 2 Case representation

**Figure 1:**
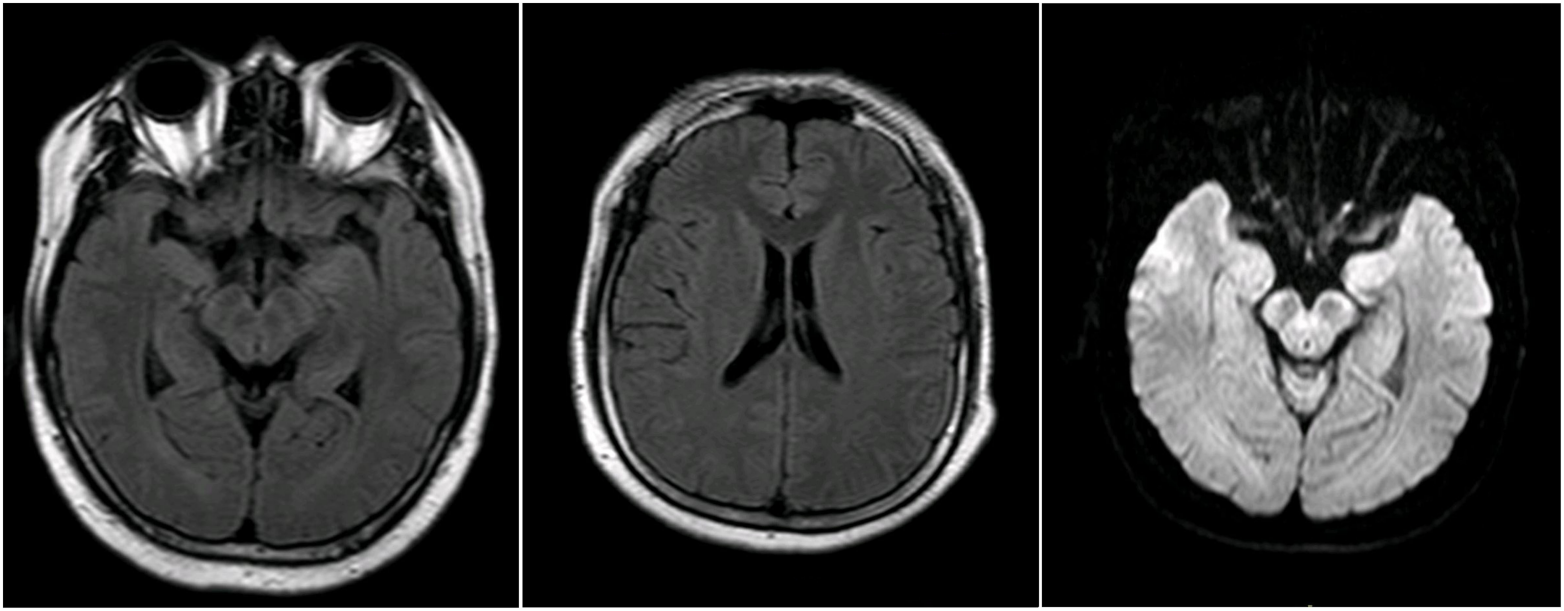
Brain MRI images of the patient.

A 30-year-old previously healthy Chinese male presented with fever and chills after catching a cold. The patient did not pay adequate attention to or appropriately manage his symptoms, leading to the progressive development of headache and nausea, along with a high body temperature of 39.2°C. Despite empirical antibiotic therapy (specific agents not documented), the patient experienced worsening headache and persistent fever, prompting transfer to another hospital. On admission, initial diagnostic evaluations revealed CSF pleocytosis characterized by 60% mononuclear cells and 40% multinucleated cells, along with elevated protein levels and reduced glucose and chloride concentrations (**Table 1**, day 0). Blood, urine, and CSF cultures yielded no positive findings. Initial brain MRI showed no abnormalities (**Figure 1**), while chest computed tomography (CT) demonstrated pleural thickening. The patient was started on broad-spectrum empiric antimicrobial therapy targeting meningitic etiologies, including ceftriaxone, ganciclovir, moxifloxacin, isoniazid, and rifampicin. Although his headache gradually improved, the fever persisted, and he was admitted to our hospital for further treatment.

**Table 1:** The CSF biochemical and cytological examinations in this case.

Time	Color of CSF supernatant	Opening Pressure (mmH_2_O)	Pro (g/L)	Glu (mmol/L)	Cl (mmol/L)	IgG index (mg/L)	Microbial cultures	CSF cytology examination					
								RBC (cells/mm³)	WBC (cells/mm³)	L (%)	M (%)	N (%)	E (%)
Day 0	Unknown	240 ↑	2.86 ↑	1.97 ↓	117 ↓	ND	Negative	/	374	Unknown	Unknown	Unknown	Unknown
Day 7	Crystal clear	200	2.05 ↑	2.4	118 ↓	ND	ND	100	110	75	3	/	22
Day 10	Crystal clear	192	1.63 ↑	2.4	119 ↓	101 ↑	Negative	/	212	84	3	/	13
Day 17	Crystal clear	210 ↑	0.62 ↑	2.8	126	29.6	Negative	450	87	91	4	3	2

CSF, cerebrospinal fluid; Opening Pressure (reference range, 60–200 mmH_2_O after eight years of age and the patient in the lateral decubitus position); Pro, protein (reference range, 0.12-0.60 g/L); Glu, glucose (reference range, 2.2-3.9 mmol/L); Cl, chloride (reference range, 120–132 mmol/L); IgG index (reference range, 0–34 mg/L); RBC, red blood cell; WBC, white blood cell (reference range, < 5 cells/mm³); L, lymphocyte; M, monocyte; N, neutrophil; E, eosinophil. ND, Not done.

On admission to our hospital, the patient exhibited a body temperature of 37.5°C, a pulse rate of 91 beats/min, a blood pressure of 134/80 mmHg, and a respiratory rate of 20/min. His family history was unremarkable. Neurological examination revealed no abnormalities except for neck stiffness. Ambulatory electromyography and cranial MRI demonstrated no significant abnormalities. Given his clinical presentation (headache and fever) and initial CSF findings (elevated CSF pressure, increased protein concentration, decreased glucose level, and CSF pleocytosis with 40% multinucleated cells), bacterial meningitis was deemed highly probable, and empirical therapy with ceftriaxone sodium was initiated. Nevertheless, differential diagnoses—including viral, tubercular, and immune-mediated encephalitis—could not be excluded, prompting further diagnostic evaluation.

His complete blood count and coagulation profile were within normal limits. Serological testing for infectious pathogens, including *Brucella*, *Mycoplasma pneumoniae*, *Chlamydophila pneumoniae*, *Legionella pneumophila*, *Coxiella burnetii*, adenovirus, respiratory syncytial virus, influenza A and B viruses, and parainfluenza virus, yielded negative results. The interferon-gamma release assay, a diagnostic tool for systemic tuberculosis infection, was also negative. In addition, nucleic acid amplification testing (Xpert MTB/RIF) and acid-fast bacilli staining of sputum samples were both negative for *Mycobacterium tuberculosis*. Routine serum screening revealed the presence of IgG antibodies against herpes simplex virus types 1 and 2 (HSV-1/HSV-2), rubella virus (RV), and cytomegalovirus (CMV).

Autoimmune screening of serum was negative for IgG, IgA, IgM, rheumatoid factor (RF), complement C3, complement C4, anti-streptolysin O (ASO), anti-keratin antibodies (AKA), anti-cyclic citrullinated peptide (CCP) antibodies, antinuclear antibodies (ANA), anti-extractable nuclear antigen (ENA) antibodies, anti-DNA antibodies, antineutrophil cytoplasmic antibodies (ANCA), and antiphospholipid antibodies.

**Figure 2:**
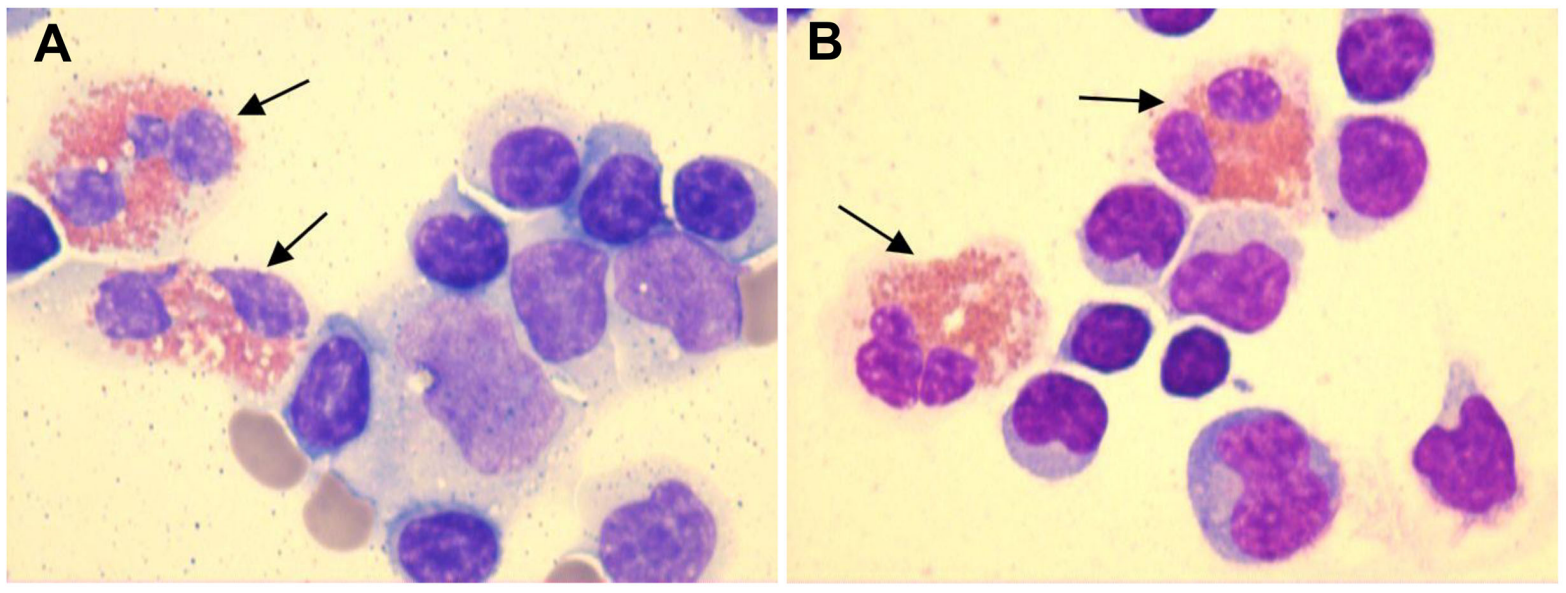
CSF smears demonstrated the presence of eosinophils (black arrows), as identified by May–Grünwald–Giemsa staining at ×1000 magnification. **(A)** CSF smear showing eosinophils, lymphocytes, monocytes, and red blood cells (**Table 1**, day 7). **(B)** CSF smear showing eosinophils and lymphocytes (**Table 1**, day 10). [The color figures were acquired using a ProgRes^®^ SpeedXTcore3 camera and ProgRes^®^ CapturePro software version 2.8.8 (Jenoptik, Germany).] .

CSF-based infectious disease screening included Alcian blue staining, acid-fast bacilli staining, microbial cultures, molecular testing for tuberculosis, and next-generation sequencing (NGS). All tests yielded negative results. Pleocytosis was observed in the CSF with a white blood cell (WBC) count of 110 cells/mm³. The CSF protein, glucose, and chloride levels were 2.05 g/L, 2.4 mmol/L, and 118 mmol/L, respectively. Five drops of CSF were pipetted into a cytofunnel and centrifuged at 113 × g for 5 min at room temperature. Air-dried smears were stained using the May–Grünwald–Giemsa method. Microscopic examination revealed a differential cell count comprising 75% lymphocytes, 3% monocytes, and 22% eosinophils (**Figure 2**; **Table 1**, day 7). Peripheral blood eosinophil levels were within the normal reference range, with a total WBC count of 6.43 × 10^9^/L and 2.0% eosinophils (reference range: 0.4%–8.0%). Systemic investigation revealed no evidence of parasitic infections, malignancy, neurosyphilis, tuberculosis, or allergic conditions. Following a 3-day course of intravenous ceftriaxone sodium, a third lumbar puncture (LP) was performed, revealing a CSF WBC count of 212 cells/mm³, composed of 84% lymphocytes, 3% monocytes, and 13% eosinophils. The CSF IgG index was elevated at 101 mg/L (upper normal limit: 34 mg/L) (**Figure 2**; **Table 1**, day 10).

Given these findings, further clinical evaluation focused on the possibility of an underlying autoimmune inflammatory disorder. CSF and/or serum testing for the following autoimmune antibodies was negative: anti-N-methyl-D-aspartate receptor (NMDAR), anti-leucine-rich glioma-inactivated 1 (LGI1), anti-α-amino-3-hydroxy-5-methyl-4-isoxazolepropionic acid 1/2 receptor (AMPA1R/AMPA2R), anti-contactin-associated protein-like 2 (CASPR2), anti-γ-aminobutyric acid receptor-B/A (GABAB/GABAA), anti-glycine receptor 1 (GlyR1), anti-glutamic acid decarboxylase (GAD65), anti-aquaporin-4 (AQP4), and anti-myelin oligodendrocyte glycoprotein (MOG). However, cell-based assays demonstrated the presence of GFAP-IgG antibodies with a titer of 1:32 in the CSF but not in the serum.

The patient received high-dose methylprednisolone pulse therapy (1,000 mg/day for 3 days). The dosage was then reduced by 50% every 3 days. When tapered to 120 mg, oral prednisone tablets (60 mg/day) were initiated, followed by gradual tapering (5 mg reduction every 2 weeks). After 3 days of 1,000 mg methylprednisolone, CSF analysis showed a WBC count of 87 cells/mm³, with 91% lymphocytes, 4% monocytes, 3% neutrophils, and 2% eosinophils. The CSF protein concentration was 0.62 g/L, with glucose and chloride levels of 2.8 mmol/L and 126 mmol/L, respectively. The IgG index was 29.6 mg/L (**Table 1**, day 17). Peripheral blood eosinophil count remained within normal limits, with a total WBC count of 6.33 × 10^9^/L and 0.06% eosinophils. The patient’s clinical symptoms, including headache and fever, resolved completely, and he was discharged. Post-discharge, he adhered strictly to the prescribed medication regimen. At the 3-month follow-up, no disease relapse was observed, and ongoing monitoring continues.

## 3 Discussion

The present case exhibited marked CSF eosinophilia, with a maximum of 22% eosinophils observed. Following detection of GFAP-IgG antibodies in the CSF, steroid pulse therapy was administered, resulting in rapid symptomatic improvement and a subsequent decrease in both the CSF WBC count and the proportion of eosinophils. No significant elevation of peripheral eosinophils was observed.

Eosinophils are not normally present in the CSF. Their presence is associated with various pathological conditions, including parasitic infections, neurosyphilis, viral, fungal, or tuberculosis infections, malignancies, allergic responses, ventriculoperitoneal shunts, and intrathecal antibiotic administration (12–16).

The patient originated from an arid area in northwest China and reported no history of consuming raw or undercooked food, including meat, fish, shrimp, or crabs. Therefore, parasitic infections were regarded as unlikely and excluded. Although serological testing revealed positive IgG antibodies for HSV-1/HSV-2, RV, and CMV, indicating prior exposure, other etiological detections in both serum and CSF were negative. Notably, NGS, a highly sensitive and reliable diagnostic tool for CNS infections, was also negative. Furthermore, all tests for *Mycobacterium tuberculosis* were negative, including interferon-gamma release assays, *rpoB* gene fragment mutation analysis, and acid-fast bacilli staining. *Cryptococcus*-specific detection via Alcian blue staining of CSF was likewise negative. In summary, this patient did not exhibit any of the aforementioned factors associated with elevated eosinophil levels.

Because eosinophilia is a typical feature of antibody-mediated disease, we considered that the increased eosinophils in the CSF might be related to an underlying autoimmune inflammatory disorder. In this case, the diagnosis of autoimmune GFAP astrocytopathy was confirmed based on the following criteria: (i) acute onset with the primary clinical syndrome being meningitis; (ii) exclusion of infectious diseases; (iii) positive detection of GFAP-IgG antibodies in CSF, which serves as a diagnostic biomarker; (iv) good response to corticosteroid therapy; and (v) CSF findings consistent with the current literature. Although no brain lesions were observed on MRI in this case, previous studies have reported that a subset of patients with autoimmune GFAP astrocytopathy may present with normal MRI findings (2, 17, 18).

To the best of our knowledge, eosinophils in CSF have been documented in a limited number of cases involving autoimmune GFAP astrocytopathy (10, 11). Yang et al. described low percentages of eosinophils in CSF without providing exact numerical values. In Patient 1, lymphocytes were 94% (CSF WBC: 225 cells/mm³). In Patient 2, lymphocytes were 97% (CSF WBC: 300 cells/mm³) and 96% (CSF WBC: 120 cells/mm³) across two separate samples. Assuming that all non-lymphocytic cells in the CSF were eosinophils, the estimated eosinophil percentages would range from 3% to 6%, with corresponding absolute counts of 13.5, 9, and 4.8 cells/mm³. Lowe et al. reported CSF eosinophil percentages ranging from 1% to 4%, with absolute counts varying between 1 and 11 cells/mm³. In our case, the maximum eosinophil percentage reached 22%, with an absolute count of 24.2 cells/mm³ (CSF WBC: 110 cells/mm³), which clearly exceeded the previously reported ranges for both relative percentage and absolute count. Although eosinophils were consistently observed across three lumbar punctures (LPs), a small number of red blood cells were detected in two of these procedures. The presence of CSF eosinophils was unlikely to be secondary to blood contamination, as true CSF eosinophil positivity was confirmed during one LP (**Table 1**, day 10), and no significant peripheral eosinophilia was observed.

The peripheral eosinophil count remained within normal limits throughout the disease progression in this case, which is consistent with the findings reported by Yang et al. In contrast, Lowe et al. observed elevated eosinophils in only a single blood test. Furthermore, a Chinese case report described a patient diagnosed with autoimmune GFAP astrocytopathy who exhibited an increased peripheral eosinophil count; however, the eosinophil count in the CSF was not specified (19).

In recent years, an increasing number of studies have reported the presence of eosinophils in CSF and histopathological analyses related to CNS autoimmune disorders. In addition to the studies reported by Yang et al. and Lowe et al., a brief case report documented the presence of eosinophils in biopsies of active lesions with autoimmune GFAP astrocytopathy (20). Previous studies have also reported eosinophils in patients diagnosed with neuromyelitis optica spectrum disorder (NMOSD) (21–23), as well as those with myelin oligodendrocyte glycoprotein antibody-associated disease (MOGAD) (24–27). Accumulating evidence has revealed the function of eosinophils in autoimmune diseases (28). Experimental models of NMOSD suggest that eosinophils play a role in the pathogenesis of neuromyelitis optica (NMO) via antibody-dependent cell-mediated cytotoxicity (ADCC) and complement-dependent cytotoxicity (CDC) mechanisms (29). Although the mechanism linking eosinophil infiltration to autoimmune GFAP astrocytopathy remains unclear, studies of CSF and serum from affected patients have revealed elevated levels of eosinophil-associated regulatory cytokines and chemokines (30, 31). Segal et al. reported increased interleukin (IL)-5 in CSF—a key cytokine regulating eosinophil growth, differentiation, and survival (30). Fu et al. identified elevated eotaxin-2 and CCL5 in serum samples (31). Eotaxin-1 (CCL11), eotaxin-2 (CCL24), and eotaxin-3 (CCL26) are primary chemotactic agents for eosinophils (28), while CCL5 promotes chemotaxis in T lymphocytes, basophils, and eosinophils within the peripheral immune system (32). Furthermore, protein–protein interaction network analysis highlighted a cluster centered on CXCL12, which was primarily associated with eosinophil, lymphocyte, and monocyte chemotaxis (31). These studies suggest that the regulation and chemotaxis of eosinophils may represent key biological processes in autoimmune GFAP astrocytopathy, with eosinophils potentially playing a significant role in disease pathogenesis.

Several limitations need to be addressed in our study. First, the patient’s tumor marker evaluation remains incomplete. Autoimmune GFAP astrocytopathy has been reported in association with neoplastic diseases (1), and eosinophils have also been linked to underlying malignancies. At the time of drafting this report, no tumors were detected in this patient. Accordingly, long-term clinical monitoring is recommended for the early detection of any potential neoplastic developments.

Second, the initial LP performed at the local hospital revealed 40% multinucleated cells in the CSF. Due to the lack of CSF cytological examination at that hospital, we were unable to determine whether eosinophils were present in the initial CSF analysis. As noted by Lowe et al., disparities in the proportion of eosinophils remain unclear and may even be underestimated in cases of autoimmune GFAP astrocytopathy. Based on the following rationale, we speculated that eosinophils were likely present in the CSF during the initial LP: (i) eosinophils are characterized by bilobular nuclei and are classified as multinucleated cells; and (ii) the proportion of eosinophils in the CSF tends to decrease as the disease progresses. Considering the possible presence of neutrophils, which are also multinucleated cells, we estimated that eosinophils in the initial CSF sample were likely between 22% and 40%.

Finally, the CSF samples from this patient were not preserved, resulting in the absence of measurements for eotaxin-1, eotaxin-2, eotaxin-3, IL-5, and associated molecules.

This case also prompted several research questions: (i) What is the incidence of eosinophilic involvement in patients with autoimmune GFAP astrocytopathy, and what proportion of CSF cells are eosinophils? (ii) Does the average percentage of serum eosinophils differ significantly between cases with and without CSF eosinophils? (iii) Is there a correlation between the CSF eosinophil percentage and clinical severity, GFAP-IgG antibody titer, or CSF WBC count?

Future studies should focus on larger patient cohorts, integrating broader clinical and laboratory parameters, as well as the measurement of eosinophil-associated regulatory cytokines and chemokines in CSF, to enhance understanding of eosinophil infiltration and the underlying pathogenic mechanisms in autoimmune GFAP astrocytopathy.

## 4 Conclusions

We presented the case of a patient diagnosed with autoimmune GFAP astrocytopathy who exhibited an elevated proportion of eosinophils in the CSF, exceeding previously reported levels in such cases, while the peripheral eosinophil count remained within normal limits. Our case suggests a potential association between eosinophils and the pathogenesis of autoimmune GFAP astrocytopathy, indicating that eosinophils may play a role in the development of this condition. Future studies should involve larger patient cohorts to systematically investigate the underlying mechanisms of eosinophil infiltration and their potential clinical implications in autoimmune GFAP astrocytopathy.

## Data Availability

The raw data supporting the conclusions of this article will be made available by the authors, without undue reservation.
